# Social determinants of health related to stay-at-home order adherence and social distancing attitudes among a diverse Deep South population

**DOI:** 10.1186/s12889-021-12093-w

**Published:** 2021-11-23

**Authors:** Jennifer L. Lemacks, Tammy Greer, Sermin Aras, Laurie Abbott, Darlene Willis, June Gipson, Mohamed O. Elasri

**Affiliations:** 1 Mississippi INBRE Telenutrition Center, The University of Southern Mississippi, Hattiesburg, MS USA; 2 School of Kinesiology and Nutrition, The University of Southern Mississippi, 118 College Drive #5142, Hattiesburg, MS 39406 USA; 3 Mississippi INBRE Community Engagement and Training Core, Hattiesburg, MS USA; 4 School of Psychology and Center for American Indian Research and Studies, The University of Southern Mississippi, Hattiesburg, MS USA; 5 College of Nursing, The Florida State University, Tallahassee, Florida, USA; 6 My Brother’s Keeper, Inc., Jackson, MS USA; 7 Center for Molecular and Cellular Biosciences, Mississippi INBRE, The University of Southern Mississippi, Hattiesburg, MS USA

**Keywords:** Social determinants of health, COVID-19, Public health, Rural health

## Abstract

**Objective:**

To describe COVID-19 related symptoms and medical care experienced in the first six months of the pandemic as well as stay-at-home order adherence, and attitudes related to COVID-19 risk and social distancing among a diverse sample of adults in the Deep South.

**Methods:**

Survey data were collected from 411 Louisiana and Mississippi residents for three weeks in June 2020 through social media.

**Results:**

Over half (52.5%) of participants who experienced COVID-19 related symptoms (with 41.5% experiencing at least one symptom) did not feel the severity of symptoms warranted seeking medical care. 91.6% of the Deep South adults visited certain places or did activities where visiting or gathering with other people was involved during stay-at-home mandates. Religiosity/spirituality, age, education, number of children in the home, attitudes related to COVID-19 risk of complications and social distancing were related to the greater/lesser likelihood of stay-at-home order adherence.

**Conclusions:**

Various cultural and contextual factors were related to stay-at-home order adherence. Understanding how social values, life stage, socioeconomic, and geographic factors influence stay-at-home order adherence would lead to more effective policy design to improve population adherence.

## Introduction

The novel coronavirus disease (COVID-19) has become a public health emergency worldwide after being declared as a pandemic by the World Health Organization in March 2020. The United States (US) is among the most-affected countries and with near six million cases and 190,000 deaths in the first six months [1]. Deep South states, such as Mississippi and Louisiana, with already existing health and social inequities were expected to bear a disproportionate burden from the pandemic. Early studies reported that African Americans had greater hospitalization rates from COVID-19 in comparison to other races but also pointed out the underlying factors such as chronic disease burden, residence in low-income areas, or occupational exposures that could result in a greater risk of COVID-19 infection [2, 3]. A recent study reported that higher perceived risk for COVID-19 was associated with greater income and younger age [4].

As of September 13, 2020, Mississippi and Louisiana reported a cumulative of 87,805 and 154,955 cases, respectively [5]. Louisiana became an epicenter among southern states as early as April and was the third highest in per capita reported cases in the US [6]. Mississippi had a more steady increase in the number of cases but had the second highest COVID-19 hospitalization rate in the nation by early June [7]. The emergence of COVID-19 cases and scientific and medical uncertainty of disease surveillance urged public health officials and state/local governments to identify and implement interventions to slow the spread of disease [8]. Non-pharmaceutical public health interventions (NPI) such as social distancing and isolation have been shown to be simple and cost-effective ways to control respiratory infections [9]. The Center for Disease Control and Prevention has also identified NPIs as one of the best ways to control pandemic illnesses when vaccines are not yet available [10]. By early April, both Mississippi and Louisiana issued stay-at-home orders and stated guidelines that included mask mandates, restrictions for non-essential businesses, and social distancing to slow the spread of COVID-19. Stay-at-home orders (referred to as “shelter” at home in Louisiana) in both states directed residents to stay at or shelter at home and limit movements outside of their homes beyond essential needs. There is still much too be learned about medical experiences related to the pandemic as well as attitudes and other factors related to stay-at-home order adherence. Additionally, it is critically to understand the factors related to state-at-home order adherence to better inform intervention and policy design. The purpose of this study was to describe COVID-19 related symptoms and medical care experienced in the first six months of the pandemic as well as stay-at-home order adherence, and attitudes related to COVID-19 risk and social distancing among a diverse sample of adults in the Deep South, specifically in the states of Louisiana and Mississippi. Additionally, we determined predictors of stay-at-home order adherence.

## Methods

### Study setting and participants

Adults 18 years of age and older who resided in Mississippi or Louisiana were recruited for the study via social media posts (i.e.., Facebook, Instagram). The posts were shared to various social media pages (i.e., Mississippi INBRE Telenutrition Center and Center for American Indian Research and Studies pages), and promoted by Mississippi INBRE Outreach Scholars and community partners. The data collection period occurred for three weeks in June 2020. The survey was developed to support the research of the MIOS summer research program. The survey was delivered online, lasted approximately 15 to 20 min, and began with an overview of the study and informed consent information. Participants were informed that the purpose of the survey was to conduct a health assessment in Mississippi and Louisiana that include questions related to demographics, standard health information, health views and attitudes, and nutrition, physical activity, and COVID-19 behaviors. Participants were also informed that the information gathered may be utilized for research purposes or by agencies and organizations to develop programs or provide resources to address community needs. Initial questions were presented to determine study eligibility based on age and state of residence. Individuals who did not live in Louisiana or Mississippi or were less than 18 years of age were excluded from participation in the study. After completing the survey, participants were offered the opportunity to accept a $5 Walmart electronic gift card. All study procedures were reviewed and approved by The University of Southern Mississippi Institutional Review Board.

### Measures

The survey included items related to nutrition, physical activity, COVID-19, and preventable chronic disease. Standard demographics and medical history information included items such as race/ethnicity, gender, educational levels, individual and household income, household size, religion and religiosity/spirituality, chronic disease information for self and family/household members. The study adopted/adapted items from Centers for Disease Control and Prevention COVID-19 Community Survey [11] to collect detailed information regarding COVID-19 related symptoms, medical care sought for COVID-19 related symptoms, COVID-19 related attitudes and social distancing/stay-at-home adherence behaviors. To assess COVID-19 related attitudes, participants selected their agreement on a 7-point Likert scale with the following six statements: 1. I have a greater risk of COVID-19; 2. I live in a neighborhood where it is difficult to socially distance myself from others; 3. It is easy for me to socially distance from family members in my home; 4. It is more important to be with my family than to socially distance from them; and 5. It will help decrease the spread of COVID-19 fi I stay away from other people, including my family. Social distancing/stay-at-home order adherence behaviors were yes/no responses for having done any of the following behaviors during the stay-at-home order time period (April 2020): 1. Gone out to a restaurant, bar, club, or other place where people gather; 2. Visit with friends, relatives, or neighbors aged 60 years or older; 3: Gone to the grocery store or pharmacy; 4: Gone to a friend, neighbor, or relative’s house (other than your own); 5. Had more than 10 friends, neighbors, or relatives over for a gathering or meal; 6. Gone to a family gathering with more than 10 people; 7. Gone to a gathering of friends with more than 10 people; and 8: Gone to a faith-based gathering. The question stem was “During the month of April 2020, have you …” and participants were asked to “select all that apply.” Other items collected and not included in this study were fatalistic attitudes related to preventable chronic disease, dietary and physical activity behaviors, peer social support for diet and physical activity, and community health values related to preventable chronic diseases.

### Statistical analysis

Descriptive and frequency data were computed for all variables and all analyses were conducted using IBM SPSS Statistics 27.0 software. To determine whether COVID-19 -related attitudes predicted social distancing/stay-at-home adherence behaviors net of the effects of demographic and psychosocial variables, we proceeded through a series of steps to determine which variables to include in the final model. First, simple correlations among demographic variables (age, education, income, the number of adults and the number of children in the household) and psychosocial variables (religiosity/spirituality, perceptions of physical health and perceptions of social-emotional support) were computed. Eight hierarchical binary logistic regression models were used to test the contributions of demographic, psychosocial and COVID-19 -related attitude variables on each of the social distance/stay-at-home order adherence variables.

## Results

### Demographics, impact of COVID-19 on employment and medical history

411 participants attempted survey completion. Of the 411 participants, 99% of participants completed all of the demographic information, 97% reported on whether they had been diagnosed with a chronic disease, 97% reported on whether or not they experienced COVID-19 symptoms, 91% reported on social support, and 90% reported on COVID-19 related attitudes and behaviors. The decrease in responses followed the order of questions in the survey with questions asked early in the survey having higher response rates compared to those answered later in the survey. Those who completed the survey (*n* = 368), were on average older (mean, M = 33.93, standard deviation, S = 14.03) compared to those who did not complete the survey (M = 28.60, S = 12.30; F (1,409) = 5.69, *p* = .02), more educated (r = .16, *p* < .01), and more likely to be married (41%) compared to non-completers (17%; Chi Square [4] = 13.02, *p* = 0.01), but not different with regard to race, employment status, or income. Demographics for the 368 participants who completed the survey are located in Table 1.

**Table 1:** Demographic descriptive data for the Deep South sample (*n* = 368)

Demographics		Mean (SD)
Age	Years	33.93 (14.03)
**Categorical Demographics**	**Category Labels**	**% (n)**
Race/Ethnicity	White	43.5 (160)
	Black or African American	30.7 (113)
	American Indian or Alaskan Native	17.9 (66)
	Hispanic/Latino	1.4 (5)
	Asian	1.1 (4)
	Two or more races	5.4 (20)
State of Residence	Louisiana	35.3 (130)
	Mississippi	64.7 (238)
Gender	Male	25.0 (92)
	Female	75.0 (276)
Education	Less than a high school degree	3.8 (14)
	A high school degree	13.0 (48)
	Some college, but not a college degree	25.5 (94)
	A 2-year or vocational degree	16.0 (59)
	A 4-year college degree or higher	41.6 (153)
Individual Annual Income	$0 to $19,999	41.6 (153)
	$20,000 to $29,999	12.5 (46)
	$30,000 to $39,999	14.1 (52)
	$40,000 to $49,999	9.2 (34)
	$50,000 to $59,999	8.7 (32)
	$60,000 or greater	11.9 (51)
Household Annual Income	$0 to $19,999	14.1 (52)
	$20,000 to $29,999	7.9 (29)
	$30,000 to $39,999	11.1 (41)
	$40,000 to $49,999	8.2 (30)
	$50,000 to $59,999	6.3 (23)
	$60,000 or greater	52.4 (193)
Children in Household	0	54.3 (200)
	1	23.6 (87)
	2	14.9 (55)
	3	4.1 (15)
	4	1.9 (7)
	5 or more	1.1 (4)
Adults in Household	0	2.7 (10)
	1	17.7 (65)
	2	47.3 (174)
	3	19.3 (71)
	4	10.1 (37)
	5 or more	3.0 (11)
Marital Status	Single	46.5 (171)
	Married	41.0 (151)
	Cohabitating	8.4 (31)
	Divorced/Separated	4.1 (15)
Employment Status	I do not work but I stay at home to care for children or elderly family members	4.6 (17)
	I do not work because I am retired	2.4 (8)
	I have a full-time position	46.5 (171)
	I have a part-time position	7.9 (29)
	I have more than one part time position	4.3 (16)
	I am a student and work part or full time	17.1 (63)
	I am a student and do not work	7.9 (29)
	I do not work and am not looking for work	3.5 (13)
	I do not work but I am looking for work	5.7 (21)
Perceptions of Physical Health	Poor	3.3 (12)
	Fair	59 (16.0)
	Good	142 (38.6)
	Very Good	119 (32.3)
	Excellent	36 (9.8)
Health Insurance Status	Has health insurance support	88.9 (327)
	No health insurance of any kind	11.1 (41)
Religion	No religious affiliation	11.4 (40)
	Baptist	36.1 (133)
	Catholic	16.0 (59)
	Methodist	5.2 (19)
	Non-denominational	16.8 (62)
	Presbyterian	1.6 (6)
	Other	8.7 (32)
	Prefer not to respond	4.6 (17)
Religiosity/Spirituality	Not religious at all	6.3 (23)
	Not too religious	17.9 (66)
	Fairly religious	45.4 (167)
	Very religious	30.4 (112)
Social & Emotional Support	Never	2.7 (10)
	Rarely	11.4 (42)
	Sometimes	20.7 (76)
	Usually	41.6 (153)
	Always	23.6 (87)

The final surveyed sample of Deep South residents (*n* = 368) was relatively young with 78.1% of respondents 45 years or younger and an age range of 18 to 79 years. Most respondents were female (75.0%), married (41.0%) or single (46.5%), had at least some college (83.1%), reported an individual annual income of $59,999 or less (86.1%), and about half reported employment in a full-time position (46.5%). About half reported zero children (54.3%) followed by one to two children (38.5%) living in the home, and one to three adults living in the household (84.3%). Most respondents were Baptist (36.1%) followed by non-denominational (16.8%) and Catholic (16.0%). Most participants (75.8%) reported that they were fairly or very religious. Table 1 provides detailed information for demographic data.

29.7% (*n* = 104) of participants reported that the COVID-19 pandemic caused them to work remotely or from home more than usual. 14.6% (*n* = 51), 18.3% (*n* = 64) and 17.7% (*n* = 62) either worked more hours than usual, worked fewer hours than usual or were not able to work, respectively. Only 5.1% (*n* = 18) had difficulty arranging childcare and 0.9% (*n* = 3) had incurred increased childcare costs. 14.3% (*n* = 50) experienced reduced income/pay and 8.3% (*n* = 29) were not paid at all.

### COVID-19 related symptoms and medical care

214 (58.2%) of the 368 participants reported not experiencing any of the five COVID-19 related symptoms between January and July 2020. Of those who reported at least one symptom (*n* = 154), the most reported symptom was a runny/stuffy nose followed by sore throat and cough. Nearly half (46.8%) of those who experienced at least one symptom went to a healthcare professional for the COVID-19 related symptom(s). Less than a quarter (18.1%) of those individuals who sought medical care for a COVID-19 related symptom were tested for COVID-19 and 48.6% were tested for influenza. The most common healthcare setting visited was a doctor’s office (41.7%) followed by the urgent care center (38.9%). Most individuals who did not seek medical care for COVID-19 related symptoms (*n* = 82) reported they did not do so because the symptoms were not considered severe enough to seek medical care (79.3%). Those who selected the “other” response indicated the following reasons for not seeking medical care: had sinus/allergy issues, avoided missing work/school, or self-medicated (data not shown). Table 2 provides complete information for quantitative data.

**Table 2:** COVID-19 related symptoms experiences and medical care sought between January and July 2020 (n = 368)

	% (n)
**Reported COVID-19 related symptoms between January and July 2020 (n = 154)**	
Fever	29.8 (46)
Cough	59.7 (92)
Sore Throat	60.4 (93)
Runny or Stuffy Nose	79.2 (122)
Difficulty breathing	20.1 (31)
**Experienced at least 1 COVID19 related symptom and saw healthcare professional for symptom (*n*** **= 72)**	
Tested for COVID-19 at healthcare visit	18.1 (13)
Received positive test result for COVID-19	15.4 (2)
Tested for influenza at healthcare visit	48.6 (35)
Received positive test result for influenza	8.6 (3)
**Location of healthcare visit for COVID-19 related symptom (*n*** **= 72)**	
Doctor’s office	41.7 (30)
Urgent care center	38.9 (28)
Telemedicine/telephone triage	8.3 (6)
Emergency room at a hospital	6.9 (5)
Health department/public health clinic	2.8 (2)
Somewhere else	1.4 (1)
**Reasons for not seeking medical care for COVID-19 related symptom (n = 82)**	
Did not feel bad enough	79.3 (65)
Distrust healthcare system	8.5 (7)
Did not have insurance	13.4 (11)
Worried about cost	11.0 (9)
Did not want to spend money	15.9 (13)
Did not have paid sick leave	9.8 (8)
No one to look after my family	2.4 (2)
Other	17.1 (14)

### COVID-19 related behaviors and attitudes

During the month of April 2020, which is the time period when both states of Mississippi and Louisiana were under a stay-at-home order, the majority of respondents (85.1%) visited a grocery store/pharmacy followed by visiting a friend, neighbor, or relative’s house (60.1%), and visited with friends, relatives or neighbors aged 60 years or older (31.4%). Only 7.5% (*n* = 31) of respondents did not visit any of the places or do any of the activities listed. Other social distancing/stay-at-home adherence behaviors are described in Table 3.

**Table 3:** Most to least common social behaviors during stay-at-home order (April 2020, *n* = 337)

	% (n)
Gone to the grocery store or pharmacy	85.1 (313)
Gone to a friend, neighbor, or relative’s house (other than your own)	60.1 (221)
Visited with friends, relatives, or neighbors aged 60 years or older	31.4 (129)
Gone out to a restaurant, bar, club, or other place where people gather	21.7 (80)
Gone to a family gathering with more than 10 people	14.1 (82)
Gone to a faith-based gathering	10.3 (38)
Had more than 10 friends, neighbors, or relatives over for a gathering or meal	8.7 (32)
Gone to a gathering of friends with more than 10 people	8.8 (38)

54.9% of participants somewhat disagreed-to-strongly disagreed that they have a greater risk of COVID-19 complications. 81.3% of participants somewhat disagreed-to-strongly disagreed that they live in a neighborhood where it is difficult to socially distance from others. 41.0% of respondents somewhat agreed-to-strongly agreed that it is more important to be with family than to social distance. 62.5% of participants somewhat agreed-to-strongly agreed that staying away from other people, including family, would help decrease the spread of COVID-19. A complete distribution of data is reported in Table 4.

**Table 4:** Likert scale distribution of attitudes related to COVID-19 risk and social distancing, n = 368

	Strongly Disagree	Disagree	Somewhat Disagree	Neither Agree nor Disagree	Somewhat Agree	Agree	Strongly Agree
**Attitude Statements**	**% (n)**						
I have a greater risk of COVID-19 complications.	28.0 (103)	26.9 (99)	5.4 (20)	13.9 (51)	10.9 (40)	8.4 (31)	6.5 (24)
I live in a neighborhood where it is difficult to socially distance myself from others.	43.8 (161)	37.5 (138)	4.3 (16)	7.6 (28)	3.0 (11)	2.4 (9)	1.4 (5)
It is easy for me to socially distance from family members in my home.	17.9 (66)	16.0 (59)	7.6 (28)	8.7 (32)	11.4 (42)	25.3 (93)	13.0 (48)
It is more important to be with my family than to socially distance from them.	9.2 (34)	15.8 (58)	8.2 (30)	25.8 (95)	14.9 (55)	15.8 (58)	10.3 (38)
It will help decrease the spread of COVID-19 if I stay away from other people including my family.	5.7 (21)	7.3 (27)	7.9 (29)	16.6 (61)	19.3 (71)	26.6 (98)	16.6 (61)

### Correlation and regression analyses

Power analysis indicated that a sample size of 300 was needed to detect between a small and medium effect size (f [2] = .10, power = .99, alpha = .05) to test for the relation between COVID-19 behaviors and the five COVID-19 attitudes over and above the explanatory power of demographic variables [5] and psychosocial variables [3]. Simple correlations among demographic and psychosocial variables revealed, at most, moderate correlations between predictor variables (See Table 5). Moderate correlations were also noted among attitudes related to COVID-19 risk and social distancing and social behaviors related to stay-at-home order adherence (See Table 6). Findings indicated that the older the participants and greater agreement that “it will help decrease the spread of COVID-19 if I stay away from other people, including my family,” the less likely participants were to report going to a restaurant, bar, club, or other place where people gather. The younger and more educated the participants, and the greater the agreement with “It is more important to be with my family than to socially distance from them,” the more likely to report having gone to a friend, neighbor, or relative’s home. The greater the importance to be with family than to socially distance from them, the more likely they were to report attending family gatherings of 10 or more people. The greater the agreement that they lived in a neighborhood where it is difficult to social distance, the more likely they were to have gone to a gather of friends with more than 10 people. The more religious the participants, the more likely they were to report having gone to a gathering with friends with more than 10 people or attended a faith-based gathering. Table 7 contains complete regression analyses results.

**Table 5:** Correlations Among Demographic Predictor Variables (n = 368)

Variable [Mean (Standard Deviation), Range]	Age [33.9 (14.0), 18–79]	Education [3.8 (1.2), 1–5]	Income [4.0 (2.5), 2–12]	Household Adults [2.3 (1.0), 1–6]	Household Children [0.8 (1.1), 0–6]	Perceptions of Health [3.3 (1.0), 1–5]	Social & Emotional Support [3.7 (1.0), 1–5]	Spirituality [3.0 (0.9), 1–4]
Age	–	.05	.54**	−.15**	−.08	−.05	.14**	.17**
Education			.29**	.02	−.11*	.14**	.07	.04
Income				−.19**	−.07	.10*	.14**	.04
Household Adults					.15**	.14**	.03	.01
Household Children						−.01	.001	−.13*
Perceptions of Health							.29**	.17**
Social & Emotional Support								.18**

* *p* < .05, ** p < .01

**Table 6:** Correlations among Attitudes related to COVID-19 Risk and Social Distancing and Social Behaviors Related to Stay-at-Home Order Adherence

	Gone out to a restaurant, bar, club, or other place where people gather	Visited with friends, relatives, or neighbors aged 60 years or older	Gone to the grocery store or pharmacy	Gone to a friend, neighbor, or relative’s house (other than your own)	Had more than 10 friends, neighbors, or relatives over for a gathering or meal	Gone to a family gathering with more than 10 people	Gone to a gathering of friends with more than 10 people	Gone to a faith-based gathering
**I have a greater risk of COVID-19 complications.**	−0.15**	−0.059	−0.078	−.117^*^	−0.067	−0.021	−0.016	−0.041
**I live in a neighborhood where it is difficult to socially distance myself from others.**	0.024	−0.033	−0.019	0.046	0.097	0.066	0.055	−0.032
**It is easy for me to socially distance from family members in my home.**	−.147^**^	−0.068	−0.038	−.116^*^	−.132^*^	−.109^*^	− 0.087	0.079
**It is more important to be with my family than to socially distance from them.**	0.084	.111^*^	0.040	.119^*^	.125^*^	.159^**^	0.090	0.052
**It will help decrease the spread of COVID-19 if I stay away from other people including my family.**	−.184^**^	−0.042	−0.025	−0.070	−.122^*^	−.168^**^	−.115^*^	−.120^*^

p < .05, ** p < .01

**Table 7:** Regression Analyses with Demographic, Psychosocial, and Attitudes Predictors of Social Behaviors related to Stay-At-Home Order Adherence

		Social behaviors related to stay-at-home order adherence							
		Gone out to a restaurant, bar, club, or other place where people gather	Visited with friends, relatives, or neighbors aged 60 years or older	Gone to the grocery store or pharmacy	Gone to a friend, neighbor, or relative’s house (other than your own)	Had more than 10 friends, neighbors, or relatives over for a gathering or meal	Gone to a family gathering with more than 10 people	Gone to a gathering of friends with more than 10 people	Gone to a faith-based gathering
		b (odds)							
**Step 1** **Demographic** **Variables**	**Age**	.97**	.99	1.01	.97**	.98	1.00	.97	.98
	**Education**	.84	1.1	1.12	1.26**	.77	.98	1.08	.81
	**Income**	.95	.94	.93	1.00	.96	.95	.95	.90
	**Household Adults**	1.1	.96	1.12	.93	1.08	.88	.77	.83
	**Household Children**	.85	.93	.91	.85	1.10	1.32	.91	1.11
	**Y intercept**	2.0	.65	3.04	2.94*	.52	.26	.46	.79
	*df* = 5	χ^2^ = 21.3**	χ^2^ = 4.6	χ^2^ = 3.4	χ^2^ = 28.0**	χ^2^ = 9.2	χ^2^ = 6.5	χ^2^ = 6.5	χ^2^ = 8.3
**Step 2** **Psychosocial Variables**	**Religiosity**	1.02	1.30	.86	.93	1.26	1.07	1.8*	3.94**
	**Health**	1.02	1.05	.84	.99	.99	1.16	.76	.95
	**Social Support**	1.16	1.02	1.29	1.02	.91	.85	.96	.73
	**Y intercept**	1.16	.27	3.57	3.44	.38	.23	.20	.04*
	*df* = 3 *df* = 8	χ^2^ = 1.5 χ^2^ = 22.8**	χ^2^ = 4.4 χ^2^ = 9.0	χ^2^ = 3.8 χ^2^ = 7.1	χ^2^ = .4 χ^2^ = 28.3**	χ^2^ = 1.3 χ^2^ = 10.5	χ^2^ = 1.6 χ^2^ = 8.1	χ^2^ = 6.3 χ^2^ = 14.2	χ^2^ = 23.4** χ^2^ = 28.3**
**Step 3** **Attitudes related to COVID-19 risk and social distancing**	**I have a greater risk of COVID-19 complications.**	.86	.98	.86	.93	.90	1.01	.88	1.02
	**I live in a neighborhood where it is difficult to social distance myself from others.**	1.06	.94	1.03	1.04	1.27	1.15	1.78*	.89
	**It is easy for me to socially distance from family members in my home.**	.89	.94	.98	.93	.86	.94	.79	1.23*
	**It is more important to be with my family than to socially distance from them.**	1.04	1.12	1.04	1.17*	1.18	1.24*	.96	1.02
	**It will help decrease the spread of COVID19 if I stay away from other people, including my family.**	.80**	1.00	.98	.95	.89	.84	1.00	.74*
	**Y intercept**	7.78	.36	6.83	5.21	.52	.22	1.11	.07
	*df* = 5 *df* = 13	χ^2^ = 21.3** χ^2^ = 44.1**	χ^2^ = 6.3 χ^2^ = 15.3	χ^2^ = 4.0 χ^2^ = 11.1	χ^2^ = 13.5* χ^2^ = 41.8**	χ^2^ = 14.5* χ^2^ = 25.0*	χ^2^ = 16.2* χ^2^ = 24.4*	χ^2^ = 8.9 χ^2^ = 23.1*	χ^2^ = 9.9 χ^2^ = 41.6*

## Discussion

This study identified COVID-19 related symptoms and medical care during the first six months of the pandemic, and described attitudes, demographic and psychosocial factors that were related to social distancing/stay-at-home adherence behaviors during statewide stay-at-home mandates. Our findings indicated that near half of participants who experienced COVID-19 related symptoms between January and July 2020 sought medical care at predominantly doctor’s offices and urgent care centers. Most of the other half did not seek medical care for symptoms because they did not feel symptoms were severe enough for medical care. Approximately two-thirds of participants reported that they disagreed that they were at greater risk of COVID-19 complications and agreed that staying away from other people would decrease the spread of COVID-19. An overwhelming majority reported that they lived in a neighborhood where it was not difficult to social distance from others. Several factors, including demographics, psychosocial and COVID-19 attitudes, were related to a greater/lessor likelihood of stay-at-home order adherence.

NPIs such as stay-at-home orders have been shown to reduce the spread of disease, including reduced rates of COVID-19 cases [12]. A current longitudinal study found that individuals living in highly populated areas made less frequent essential trips to places like the grocery store or pharmacy and were more likely to adhere to social distancing guidelines [13]. Louisiana and Mississippi are largely rural states, and most participants did visit a grocery store/pharmacy during the stay-at-home order period. It should be noted that our sample was composed of a greater proportion of women, who could be caretakers of their home and responsible for the food and other household necessities. Another study in Bangladesh women was similar where 89.1% of 2424 respondents reported leaving the house for “shopping necessities” during the over two-month stay-at-home order [14]. Additionally, near half of our sample reported changes to employment that would worsen socioeconomic conditions. While stay-at-home orders may reduce or slow disease incidence, economical and psychological impacts have also been reported at individual and community levels among other populations [14, 15]. This is important to consider, especially in the Deep South, where underserved populations already experience disparities in many areas, which could be exacerbated by stay-at-home orders.

Most participants reported as fairly/very religious/spiritual and some type of religious affiliation. This region of the country is among the highest for church attendance compared to other states and regions of the US [16]. Other research has shown that attending places of worship within two weeks prior to testing positive for COVID-19 had higher likelihood of testing positive for COVID-19 [17]. Our research shows that religiosity/spirituality did increase the likelihood of having attended a faith-based gathering during the stay-at-home order. This could be a unique contributor to the spread of COVID-19 in this region.

Attitudes about staying away from other people to prevent COVID-19 and the importance of family over social distancing along with age, was associated with the likelihood of going where other people gather (restaurants/bars/clubs) and to faith-based gatherings. Younger adults and disagreement with staying away from other people would help decrease the spread of COVID-19 were more likely to have gone to gathering places; younger adults were also more likely to have gone to a gathering of friends with more than 10 people. Research has reported that COVID-19 transmission was highest in younger adults later on in the pandemic (June to August) compared to earlier on (January to May) [18]. Additionally, a very large study of over 30,000 people in the UK (the CORSAIR study) found that non-adherence to self-isolation behaviors was associated with younger age groups [19]. This could be attributed to public health messages that older/elderly adults were at a greater risk of COVID-19 complications compared to younger populations.

Those who placed greater value on being with family than social distancing were more likely to go to a family gathering of more than 10 people and to a friend, neighbor, or relative’s house. Prior research has shown that individuals who tested positive for COVID-19 reported significantly more close contacts with a person with known COVID-19 within the two weeks prior to being tested compared to those who tested negative for COVID-19 [20]. Of those that had a close contact with an individual who tested positive for COVID-19, “family” was the most commonly reported relationship of the close contact with COVID-19, accounting for about 50% of the responses, followed by “work colleague” and “friend.” [20]

The reported results highlight unique cultural and contextual factors that may influence stay-at-home order adherence and possibly other NPIs. During a similar time period, the first two weeks of May 2020, 77.4, 83.0 and 84.6% of a US, Los Angeles, and New York City sample, respectively, reported no contact with anyone outside of their home as a COVID-19 mitigation strategy [21]. Similarly, 75.1 to 77.6% of those samples reported always avoiding groups of 10 or more people [21]. This is starkly different from our results where only 7.5% of our sample did not visit any of the places or do any of the listed activities where visiting or gathering with other people was involved. As mentioned earlier, there may be geographic attributions to stay-at-home order adherence, in addition to socioeconomic and cultural considerations. However, the CORSAIR study reported that going to the grocery store/pharmacy was one of the reasons for not self-isolating when presented with possible COVID-19 symptoms [19]. This study also reported that feeling better was another reason for not self-isolating, which was similar to our results.

Study strengths include a respectable sample size. While the sample size was adequate for our purposes and was diverse in terms of race/ethnicity, it does not necessarily represent the entire population of Louisiana and Mississippi. Compared to 2019 US Census data for the states of Louisiana and Mississippi [22, 23], our sample had approximately 24% more women, 11% less individuals with only a high school education, 17% more American Indian/Alaskan Natives, and 16% less Whites. The electronic survey was an ideal data collection method amidst a pandemic and our approach leveraged widely used social platforms. However, bias may be introduced due to social media use and internet access required of our approach. Other limitations include biases associated with self-report measures. We also do not know the frequency of the social behaviors exhibited during the stay-at-home order, which could influence results.

## Conclusions

As the US continues to manage the pandemic and identifies strategies to mitigate the transmission of COVID-19, cultural and contextual factors need to be considered to identify state level strategies for most effective NPI implementation. As future stay-at-home/lockdown recommendations are considered by policy makers, understanding how social values, life stage, socioeconomic, and geographic factors influence stay-at-home order adherence would lead to more effective policy design to improve population adherence.

## Data Availability

The datasets used and/or analyzed during the current study are available from the corresponding author on reasonable request.

## References

[1] Trends in Number of COVID-19 Cases in the US Reported to CDC, by State/Territory. Atlanta, GA: Centers for Disease Control and Prevention. https://covid.cdc.gov/covid-data-tracker. Accessed Oct 30, 2020.

[2] Price-Haywood EG, Burton J, Fort D, Seoane L (2020). Hospitalization and mortality among Black patients and white patients with COVID-19. N Engl J Med.

[3] Gold JAW, Wong KK, Szablewski CM, et al. Characteristics and Clinical Outcomes of Adult Patients Hospitalized with COVID-19 - Georgia, March 2020. *MMWR Morb Mortal Wkly Rep.* 2020;69(18):545–550. Published 2020 May 8. doi:10.15585/mmwr.mm6918e1 10.15585/mmwr.mm6918e1 PMC7737948 32379729

[4] Masters NB, Shih SF, Bukoff A, et al. Social distancing in response to the novel coronavirus (COVID-19) in the United States. *PLoS One.* 2020;15(9):e0239025. Published 2020 Sep 11. doi:10.1371/journal.pone.0239025 10.1371/journal.pone.0239025 PMC7485770 32915884

[5] COVID-19 Dashboard by the Center for Systems Science and Engineering. Johns Hopkins University website. https://coronavirus.jhu.edu/map.html. Accessed Oct 30, 2020.

[6] Daigle A. Coronavirus cases grew faster in Louisiana than anywhere else in the world: UL study. TheAcadiana. https://www.theadvocate.com/acadiana/news/coronavirus/article_94494420-6d4b-11ea-ac42-ff7dd722c084.html. .

[7] Hensley E. Mississippi COVID-19 hospitalization rate ranks second in nation amid state data blackout. Mississippi Today. https://mississippitoday.org/2020/06/22/mississippi-covid-19-hospitalization-rate-ranks-second-in-nation-amid-state-data-blackout/. .

[8] Implementation of mitigation strategies for communities with local COVID-19 transmission. Atlanta, GA: CDC. https://www.cdc.gov/coronavirus/2019-ncov/community/community-mitigation.html. Accessed Oct 30, 2020.

[9] Haug N, Geyrhofer L, Londei A, Dervic E, Desvars-Larrive A, Loreto V, Pinior B, Thurner S, Klimek P (2020). Ranking the effectiveness of worldwide COVID-19 government interventions. Nat Hum Behav.

[10] Community NPIs: Flu prevention in community settings. Atlanta, GA: CDC https://www.cdc.gov/nonpharmaceutical-interventions/community/index.html.

[11] CDC COVID-19 Community Survey Question Bank. Bethesda, MD: National Library of Medicine, NIH. https://cde.nlm.nih.gov/formView?tinyId=Kcceysolt. Published 2020. Accessed Oct 30, 2020.

[12] Gao S, Rao J, Kang Y, Liang Y, Kruse J, Dopfer D, Sethi AK, Mandujano Reyes JF, Yandell BS, Patz JA (2020). Association of mobile phone location data indications of travel and stay-at-home mandates with COVID-19 infection rates in the US. JAMA Netw Open.

[13] Hamidi S, Zandiatashbar A (2021). Compact development and adherence to stay-at-home order during the COVID-19 pandemic: a longitudinal investigation in the United States. Landsc Urban Plan.

[14] Williams SN, Armitage CJ, Tampe T, Dienes K. Public perceptions and experiences of social distancing and social isolation during the COVID-19 pandemic: a UK-based focus group study. *BMJ Open*. 2020;10(7):e039334. doi: 10.1136/bmjopen-2020-039334. Published 2020 Jul 20. doi:10.1136/bmjopen-2020-039334 10.1136/bmjopen-2020-039334 PMC7387310 32690752

[15] Hamadani JD, Hasan MI, Baldi AJ, Hossain SJ, Shiraji S, Bhuiyan MSA, Mehrin SF, Fisher J, Tofail F, Tipu SMMU, Grantham-McGregor S, Biggs BA, Braat S, Pasricha SR (2020). Immediate impact of stay-at-home orders to control COVID-19 transmission on socioeconomic conditions, food insecurity, mental health, and intimate partner violence in Bangladeshi women and their families: an interrupted time series. Lancet Glob Health.

[16] Attendance at religious services by state. Pew Research Center website. https://www.pewforum.org/religious-landscape-study/compare/attendance-at-religious-services/by/state/. Accessed Nov 11, 2020.

[17] Clipman SJ, Wesolowski AP, Gibson DG, Agarwal S, Lambrou AS, Kirk GD, et al. Rapid real-time tracking of non-pharmaceutical interventions and their association SARS-CoV-2 positivity: The COVID-19 Pandemic Pulse Study [published online ahead of print, 2020 Sep 2]. Clin Infect Dis. 2020, 2021:ciaa1313. 10.1093/cid/ciaa1313. 10.1093/cid/ciaa1313 PMC7499535 32877921

[18] Boehmer TK, DeVies J, Caruso E, van Santen KL, Tang S, Black CL, et al. Changing age distribution of the COVID-19 pandemic - United States, May–August 2020. MMWR Morb Mortal Wkly Rep. 2020;69(39):1404–1409. Published 2020 Oct 2. doi:10.15585/mmwr.mm6939e1 10.15585/mmwr.mm6939e1 PMC7537561 33001872

[19] Smith LE, Potts HW, Amlȏt R, Fear NT, Michie S, Rubin GJ. Adherence to the test, trace and isolate system: results from a time series of 21 nationally representative surveys in the UK (the COVID-19 Rapid Survey of Adherence to Interventions and Responses [CORSAIR] study). medRxiv. Published online September 18, 2020:2020.09.15.20191957. doi:10.1101/2020.09.15.20191957

[20] Fisher KA, Tenforde MW, Feldstein LR, Lindsell CJ, Shapiro NI, Files DC, et al. Community and close contact exposures associated with COVID-19 among symptomatic adults ≥18 years in 11 outpatient health care facilities - United States, July 2020. MMWR Morb Mortal Wkly Rep. 2020;69(36):1258–1264. Published 2020 Sep 11. doi: 10.15585/mmwr.mm6936a5. 10.15585/mmwr.mm6936a5 PMC7499837 32915165

[21] Czeisler MÉ, Tynan MA, Howard ME, Honeycutt S, Fulmer EB, Kidder DP, et al. Public Attitudes, behaviors, and beliefs related to COVID-19, stay-at-home orders, nonessential business closures, and public health guidance — United States, New York City, and Los Angeles, May 5–12, 2020. MMWR Morb Mortal Wkly Rep. 2020;69(24):751–758. Published 2020 Jun 19. doi:10.15585/mmwr.mm6924e1 10.15585/mmwr.mm6924e1 PMC7302477 32555138

[22] U.S. Census Bureau QuickFacts: Louisiana. Accessed May 27, 2021. https://www.census.gov/quickfacts/LA

[23] U.S. Census Bureau QuickFacts: Mississippi. Accessed May 27, 2021. https://www.census.gov/quickfacts/MS

